# Generalised exponential-Gaussian distribution: a method for neural reaction time analysis

**DOI:** 10.1007/s11571-022-09813-2

**Published:** 2022-05-17

**Authors:** Fernando Marmolejo-Ramos, Carlos Barrera-Causil, Shenbing Kuang, Zeinab Fazlali, Detlef Wegener, Thomas Kneib, Fernanda De Bastiani, Guillermo Martinez-Flórez

**Affiliations:** 1 Centre for Change and Complexity in Learning, University of South Australia, Adelaide, 5000 Australia; 2 Facultad de Ciencias Exactas y Aplicadas, Instituto Tecnológico Metropolitano -ITM, Medellín, 050034 Colombia; 3 Institute of Psychology, Chinese Academy of Sciences, Beijing, 100101 China; 4 School of Cognitive Sciences, Institute for Research in Fundamental Sciences (IPM), Tehran, Iran; 5 Department of Psychiatry, Division of Integrative Neuroscience, Columbia University and the New York State Psychiatric Institute, New York, USA; 6 Brain Research Institute, Center for Cognitive Science, University of Bremen, Bremen, Germany; 7 Campus Institute Data Science (CIDAS) and Chair of Statistics, Georg-August-Universität Göttingen, Göttingen, Germany; 8 Statistics Department, Federal University of Pernambuco, Recife, Pernambuco Brazil; 9 Departamento de Matemáticas y Estadística, Facultad de Ciencias, Universidad de Córdoba, Córdoba, 2300 Colombia; 10 Programa de Pós-Graduação em Modelagem e Métodos Quantitativos, Universidade Federal do Ceará, Fortaleza, Brazil

**Keywords:** Exponential Gaussian distribution, Reaction times, Neuronal response latency, Cognitive neuroscience, Generalised additive models for location, Scale and shape

## Abstract

Reaction times (RTs) are an essential metric used for understanding the link between brain and behaviour. As research is reaffirming the tight coupling between neuronal and behavioural RTs, thorough statistical modelling of RT data is thus essential to enrich current theories and motivate novel findings. A statistical distribution is proposed herein that is able to model the complete RT’s distribution, including location, scale and shape: the generalised-exponential-Gaussian (GEG) distribution. The GEG distribution enables shifting the attention from traditional means and standard deviations to the entire RT distribution. The mathematical properties of the GEG distribution are presented and investigated via simulations. Additionally, the GEG distribution is featured via four real-life data sets. Finally, we discuss how the proposed distribution can be used for regression analyses via generalised additive models for location, scale and shape (GAMLSS).

## Introduction

The brain is a system that consists of millions of thousands of neurons designed to perceive and respond to external stimulus in a highly nonlinear and complex way. Reaction times, broadly defined as the time lapse between the presentation of a stimulus and a response to it, have been proven a ubiquitous metric extensively used in experimental psychology, cognitive neuroscience, psychophysiology and behavioural neuroscience to explain the mechanisms supporting higher- and lower-order cognitive processes. Research suggests there is a tight coupling between neuronal RTs and behavioural RTs (see Palmer et al. 2007; Galashan et al. 2013; Mukamel and Fried 2012; Levakova et al. 2015) and that neural processing, signal transmission, and decision processes are bundled within that time lapse (see Voelker et al. 2017; see Figure 1 in Commenges and Seal (1986) for a representation of the association between stimulus, neuronal RTs, and behavioural RT). The importance of investigating RTs has been demonstrated via computational, human- and animal-based behavioural approaches. From a computational perspective, for example, it has been shown that behavioural RTs can be used as biomarkers for characterising neurological diseases (Maia and Kutz 2017). From a human behavioural perspective, evidence shows that RTs can indeed allow differentiation between certain brain diseases. For example, Jahanshahi et al. (1993) showed that patients with Parkinson’s disease, Huntington’s disease and cerebellar disease exhibit different average RTs, and Osmon et al. (2018) demonstrated the clinical advantage of examining the distribution of Attention Deficit/Hyperactivity and neurotypical patients’ RTs. Animal-based research has shown how and when specific sets of neurons fire to selected stimuli under highly controlled experimental settings (e.g. Sun et al. 2019; Luna et al. 2005; Múnera et al. 2012; Veit et al. 2014).

Various distributions including the Inverse-Gaussian (or Wald), Weibull, Log-Normal, Weibull-Gaussian, and Gamma have been used to fit behavioural and neural reaction time data. For example, Palmer et al. (2007) used the Weibull distribution, Leiva et al. (2015) used the Birnbaum–Saunders distribution, and Seal et al. (1983) used the Gamma distribution. Other distributions have recently been proposed for modelling RT data. For example, Tejo et al. (2018, 2019), and Martínez-Flórez et al. (2020) have put forward the shifted Birnbaum–Saunders, shifted Gamma distributions, and Exponential-Centred Skew-Normal distributions, respectively, as good fits to RTs. For example, Osmon et al. (2018) fitted the participants’ RTs with the three-parameter Johnson’s SU distribution,^1^ and Foroni et al. (2020) found the four-parameter Sinh-Arcsinh distribution gave the best fit for simple choice RTs. However, the distribution most commonly used to fit RT data is the Exponential Gaussian (also known as Exponentially Modified Gaussian or Ex-Gaussian distribution; here referred to as EG). This is a three-parameter distribution that can fit the data’s location, scale and rightward exponential shape. The EG hence shows a positive skew, which is the canonical shape of both neuronal and behavioural RTs [e.g. see Figure 4 in Hanes and Schall (1996), Figure 2 in Hauser et al. (2018), and Figure 2 in Mormann et al. (2008) for neuronal RT shapes, Figure 7 in Osmon et al. (2018) and Figures 3C, 4C, and 5B in Fischer and Wegener (2018) for behavioural RT shapes]. Although flexible enough to fit the typical positive skew distribution of RTs, the EG cannot fit RTs that exhibit normal-like or even negative skew. Normal- and negative-like shapes have been reported for rate RTs (reciprocal RTs, i.e. 1/RT) [see Figure 2A in Harris et al. (2014)] and recognition (go/no-go) RTs [see Figure 2 in Limongi et al. (2018)], respectively.

In this article, a four-parameters distribution called Generalised Exponential Gaussian is proposed. Similar to the EG distribution, the proposed distribution can fit positively skewed RT shapes but it has the advantage of fitting Gaussian-like and negatively skewed RT shapes. The article unfolds as follows: first, statistical arguments in favour of asymmetric distributions are presented; second, the properties of the EG are outlined; third, the details of the proposed distribution are described; fourth, the results of a computer simulation examining the properties of the proposed distribution are reported; fifth, the proposed distribution is illustrated via four data sets; sixth, generalised additive models for location, scale and shape (GAMLSS) are briefly presented, as this is the only existing regression framework suitable to model regression data in a fully distributional fashion; and finally, the statistical and practical implications of the proposed distribution are discussed in relation to GAMLSS.

## Asymmetric distributions as better alternatives to model non-normal data

Most statistical analyses featured in published research rely on techniques that assume, among other things, that data follow a normal distribution. In practice, however, data tend to follow non-normal shapes [see Bono et al. (2017)]. When faced with non-normal distributions, the common approach is to transform the numeric variables. Although transformations can indeed be successful, they bring challenges in relation to the interpretability of the new metric and back-transformation (Marmolejo-Ramos et al. 2015; Pek et al. 2018; Vélez et al. 2015). Specifically, it is not always possible to find a back-transformation that enables interpretation of the parameter estimate, and this issue is more pronounced when there are several variables with different transformations [see Azzalini and Capitanio (1999)]. Distributions more flexible than the traditional Gaussian have been proposed to overcome these challenges. These newly proposed distributions enable data with different degrees of asymmetry and kurtosis to be tackled. Some of these distributions are the Skew-Normal [SN; Azzalini (1985)], Power-Normal [PN; Durrans (1992); Pewsey et al. (2012)], and Skew-Normal Alpha-Power [here called SNAP for short; Martínez-Flórez et al. (2014)].

The SN model is defined by the probability density function (PDF)

1 $$\begin{aligned} \varphi (z;\lambda )=2\phi (z)\Phi (\lambda z), \quad z\in {\mathbb{R}}, \end{aligned}$$

where $\phi$ and $\Phi$ are the density and distribution functions, respectively, of the standard normal distribution, and $\lambda$ is a skewness parameter. This distribution is denoted by $Z\sim {SN(\lambda )}$ and, in addition to the work by Azzalini (1985), it has been extensively studied by Henze (1986), Chiogna (1998), Gómez et al. (2007) and Pewsey et al. (2012).

Alternatively, Lehmann (1953) proposed a family of distributions with PDF $F(z;\alpha )=\{F(z)\}^{\alpha -1}$, where *F* is a distribution function with $z\in {\mathbb{R}}$, and $\alpha \in \mathbb{Z}^+$. In general terms, this distribution is generated from the distribution of the maximum of the sample. This model is known in the literature as Lehmann’s alternative model and is widely discussed by Gupta and Gupta (2008).^2^

The PN model, denoted by $Z\sim PN(\alpha )$ and introduced by Durrans (1992), has the following PDF:

2 $$\begin{aligned} f(z,\alpha )=\alpha \phi (z)\left\{ \Phi (z)\right\} ^{\alpha -1},\quad z \in {\mathbb{R}}, ~~\alpha \in \mathbb{R}^+, \end{aligned}$$

where $\phi$ and $\Phi$ are the density and distribution functions, respectively, of the standard normal distribution and $\alpha$ is a shape parameter. This distribution has multiple applications in cases where data cannot be handled through normal distributions and, instead, data present high or low asymmetry and/or kurtosis.

Martínez-Flórez et al. (2014) proposed the SNAP distribution ($SNAP(\lambda ,\alpha )$), a more flexible extension of the previous two distributions. This distribution not only contains the SN and PN distributions as special cases, but it also includes the normal distribution. The PDF of the SNAP distribution is as follows:

3 $$\begin{aligned} f(z,\alpha )=\alpha \phi _{SN}(z;\lambda )\left\{ \Phi _{SN}(z;\lambda )\right\} ^{\alpha -1}, \quad z, \lambda \in {\mathbb{R}}, ~~\alpha \in \mathbb{R}^+. \end{aligned}$$

The SN, PN, and SNAP distributions illustrate the versatility of asymmetric and generalised distributions for fitting non-Gaussian data (Table 1 provides a summary of key aspects of these distributions). Although it could be argued that these distributions provide good-enough fits to RT data, they have not been investigated in the context of RT experiments. The EG distribution, however, is the most commonly used distribution to fit RTs obtained in (neuro)psychological experiments [see Dawson (1988)].

**Table 1:** Summary of the asymmetric distributions skew-normal (SN), power-normal (PN), and alpha-power skew-normal (SNAP)

Distribution	PDF	CDF	Mean	Median	Skewness	Kurtosis
$SN(\lambda )$	$2\phi (z)\Phi (\lambda z)$	$\Phi (z)-2T(z;\lambda )$	$\mu +\sqrt{\frac{2}{\pi }}\sigma \frac{\lambda }{\sqrt{1+\lambda ^2}}$	$\mu +\sigma \Phi _{SN}^{-1}(0.5;\lambda )$	$[-0.9953,0.9953]$	[3, 3.8692]
$PN(\alpha )$	$\alpha \phi (z)\{\Phi (z)\}^{\alpha -1}$	$\{\Phi (z)\}^\alpha$	$\mu +\alpha \sigma \int _0^1\Phi ^{-1}(u)u^{\alpha -1}du$	$\mu +\sigma \Phi ^{-1}(0.5^{1/\alpha })$	$[-0.6115,0.9007]$	[1.7170, 4.3556]
$SNAP(\lambda , \alpha )$	$\alpha \phi _{SN}(z;\lambda )\{\Phi _{SN}(z;\lambda )\}^{\alpha -1}$	$\{\Phi (z)-2T(z;\lambda )\}^\alpha$	$\mu +\alpha \sigma \int _0^1\Phi _{SN}^{-1}(u;\lambda )u^{\alpha -1}du$	$\mu +\sigma \Phi _{SN}^{-1}(0.5^{1/\alpha };\lambda )$	$[-1.4676,0.9953]$	[1.4672, 5.4386]

$\phi (\cdot )$ and $\Phi (\cdot )$ denote the PDF and CDF of the Normal distribution; $\phi _{SN}(\cdot )$ and $\Phi _{SN}(\cdot ;\lambda )$ are the PDF and CDF of the standard Skew-Normal distribution; $\Phi ^{-1}(\cdot )$ and $\Phi _{SN}^{-1}(\cdot ;\lambda )$ denote the inverse functions of $\phi _{SN}(\cdot )$ and $\Phi _{SN}(\cdot ;\lambda )$ respectively, and $T(z;\lambda )$ is the Owen’s T function $z=\frac{x-\mu }{\sigma }$. As skewness and kurtosis do not have closed-form expressions, these are estimated numerically by integrating over the first, second, third, and fourth moments

## The Ex-Gaussian distribution and its statistical properties

This probability model was introduced by Hohle (1965) through the convolution of two independent random variables; the Normal and Exponential PDFs. This distribution was conceived between 1956 and 1963 (Christie and Luce 1956) while searching for a model that could represent the disjunctive structure of RTs. That is, the RT is composed of an exponentially distributed decision time, plus a variable time or motor RT. After studying the cumulative distribution function (CDF) of the RT for different intensities of auditory stimuli, McGill (1963) concluded that the variable had an exponential form with similar constant times. This led to the assumption that at least one component of the total RT had an exponential distribution. Given that the time constants implied by the curves appeared to be almost independent of the stimulus intensity, McGill (1963) assumed that this component was the time required for the motor response, while the other component was assumed to be the time taken to make a decision. Specifically, the EG’s exponential distribution component has a constant average response $\tau$ (Luce 1986; McGill 1963) while the remaining part of the EG follows a normal distribution $N(\mu ,\sigma ^{2})$ (Hohle 1965). These statistical properties of the EG distribution are described in more detail in “Appendix” [see also section 13.3.2.1 in Rigby et al. (2020)].

## The generalised exponential-Gaussian distribution

Based on the distribution of the sample’s maximum, Lehmann (1953) proposed the family of distributions $\mathbb{F}_F=\{F(x)\}^\alpha$ where $F(\cdot )$ is an absolutely continuous distribution function and $\alpha$ is a rational number. In the context of hydrology, Durrans (1992) extended this model to a family of distributions $\mathbb{G}_F=\{F(x)\}^\alpha$ where $F(\cdot )$ is a distribution function and $\alpha \in {\mathbb{R}}^+$. This family of distributions has PDF $g(x)=\alpha f(x)\{F(x)\}^{\alpha -1}$ where $f=dF$ denotes the distribution of fractional order statistics [see also Stigler (1977)]. When $F=\Phi$ (i.e. the Normal distribution’s CDF), it is known as the generalised normal distribution. This is a flexible distribution that can be conceived as an extreme values distribution given that $\alpha \in {\mathbb{R}}^+$. Also, when $\alpha \in \mathbb{N}$, this distribution can fit data with positive or negative skews. Finally, when $\alpha =1$, the original distribution’s PDF $f(\cdot )$ is obtained.

Following the above-mentioned work by Durrans (1992), a generalisation of the distribution of the maximum in the $f_{EG}(\cdot )$ is proposed by considering an $\alpha -$fractional order statistic where $\alpha \in {\mathbb{R}}^+$. That is, the goal is to extend the EG distribution by incorporating a new parameter that controls the skewness and kurtosis of the distribution, i.e. the distribution’s shape. As the new distribution has skewness and kurtosis values above and below those possible by the original $f_{EG}(\cdot )$, it is hence much more flexible than the traditional EG distribution in accommodating skewness and kurtosis.

**Fig. 1:**
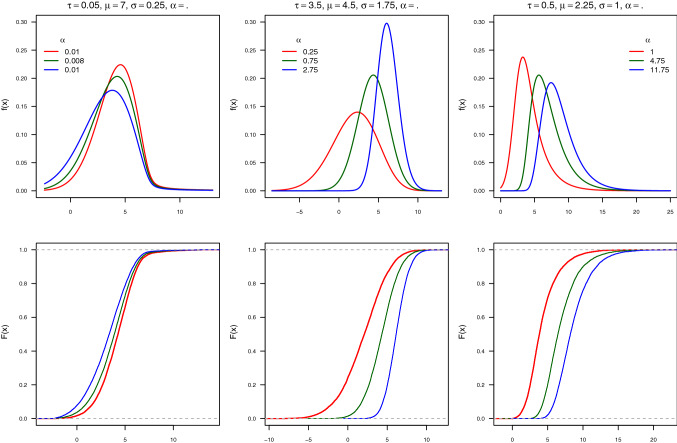
Probability density (first row) and cumulative distribution functions (second row) for values of $\tau ,\mu ,\sigma$, and $\alpha$ of the GEG distribution. Three negatively skewed, symmetric, and positively skewed shapes are shown in the first, second, and third columns respectively

According to the results given by Durrans (1992) and Pewsey et al. (2012), the exponential extension of the EG distribution is given by the following PDF:

4 $$\begin{aligned} \varphi (x)&= \frac{\alpha }{\tau }e^{-\frac{x-\mu }{\tau }+\frac{\sigma ^2}{2\tau ^2}}\Phi \left( \frac{x-\mu }{\sigma } -\frac{\sigma }{\tau }\right) \nonumber \\&\quad \left[ \Phi \left( \frac{x-\mu }{\sigma }\right) -e^{-\frac{x-\mu }{\tau } +\frac{\sigma ^2}{2\tau ^2}}\Phi \left( \frac{x-\mu }{\sigma }-\frac{\sigma }{\tau }\right) \right] ^{\alpha -1} \end{aligned}$$

such that $\alpha \in {\mathbb{R}}^+$ is a shape parameter that controls skewness and $\tau$ regulates kurtosis; $\mu$ and $\sigma$ are the location and scale parameters respectively (such that $\sigma >0$ and $-\infty<\mu <\infty$). This distribution is called from here on the Generalised Exponential Gaussian (GEG) distribution and it is denoted by $X\sim GEG(\tau ,\mu ,\sigma ,\alpha )$. Note that when $\alpha =1,$ the GEG meets the EG distribution. On the other hand, when $\tau \rightarrow 0$, then $\mathbb{E}(X)\rightarrow \mu , ~~Var(X)\rightarrow \sigma ^{2},~~skew\rightarrow 0\quad \text {and}\quad kurt\rightarrow 3$; i.e. $EG(\tau ,\mu ,\sigma ^{2})\rightarrow N(\mu ,\sigma ^{2})$. Additionally, when $\tau \rightarrow 0$ and $\alpha \ne 1$, the GEG distribution converges to the PN distribution; i.e. $GEG(\tau ,\mu ,\sigma ,\alpha )\rightarrow PN(\mu ,\sigma ,\alpha )$. Figure 1 displays some of the shapes the GEG distribution can take.

The CDF of the random variable $X\sim GEG(\tau ,\mu ,\sigma ,\alpha )$ is given by:

5 $$\begin{aligned} F_{GEG}(x)=\left[ \Phi \left( \frac{x-\mu }{\sigma }\right) -e^{-\frac{x-\mu }{\tau } +\frac{\sigma ^2}{2\tau ^2}}\Phi \left( \frac{x-\mu }{\sigma }-\frac{\sigma }{\tau }\right) \right] ^{\alpha } \end{aligned}$$

To generate a random variable with GEG distribution, a uniform random variable *U* in (0,1) should be used. So, letting

$$\begin{aligned} u=[F_{EG}(x; \tau ,\mu ,\sigma )]^\alpha , \end{aligned}$$

then

$$\begin{aligned} u^{1/\alpha }=F_{EG}(x; \tau ,\mu ,\sigma ) \end{aligned}$$

from where it follows that

$$\begin{aligned} x=F_{EG}^{-1}(u^{1/\alpha }; \tau ,\mu ,\sigma ), \end{aligned}$$

where $F_{EG}^{-1}(\cdot ;\tau ,\mu ,\sigma )$ is the inverse function of the EG distribution with parameters $\tau ,\mu ,\sigma$, which is available in the R packages emg and gamlss.dist (via the function ‘exGAUS’).

The survival, inverse risk, and the hazard functions of the GEG distribution are:

6 $$\begin{aligned} S_{GEG}(x)&= 1-\left[ \Phi \left( \frac{x-\mu }{\sigma }\right) -e^{-\frac{x-\mu }{\tau }+\frac{\sigma ^2}{2\tau ^2}}\Phi \left( \frac{x-\mu }{\sigma }-\frac{\sigma }{\tau }\right) \right] ^{\alpha },\nonumber \\&\quad r_{GEG}(t)=\frac{f_{GEG}(x)}{F_{GEG}(x)}=\alpha r_{EG}(t), \end{aligned}$$

7 $$\begin{aligned} h_{GEG}(t)&= \alpha \frac{e^{-\frac{x-\mu }{\tau }+\frac{\sigma ^2}{2\tau ^2}}\Phi \left( \frac{x-\mu }{\sigma }-\frac{\sigma }{\tau }\right) \left[ \Phi \left( \frac{x-\mu }{\sigma }\right) -e^{-\frac{x-\mu }{\tau }+\frac{\sigma ^2}{2\tau ^2}}\Phi \left( \frac{x-\mu }{\sigma }-\frac{\sigma }{\tau }\right) \right] ^{\alpha -1}}{\tau \left( 1-\left[ \Phi \left( \frac{x-\mu }{\sigma }\right) -e^{-\frac{x-\mu }{\tau }+\frac{\sigma ^2}{2\tau ^2}}\Phi \left( \frac{x-\mu }{\sigma }-\frac{\sigma }{\tau }\right) \right] ^{\alpha } \right) } \end{aligned}$$

where $r_{EG}(\cdot )$ is the inverse risk function of the EG distribution defined in Eq. (11 in “Appendix”). This entails the inverse risk function of the GEG distribution being directly proportional to the previous function, and in the same way, they are intervals where $r_{GEG}$ grows or decreases. Some properties of the exponentiated generalized class of distributions can be found in Cordeiro et al. (2013). Details regarding the GEG distribution’s moments, log-likelihood function, score function, and information matrices are presented in “Appendix”.

## A simulation-based assessment of the generalised exponential-Gaussian distribution

A simulation was carried out to investigate the maximum likelihood estimates (MLEs) of the GEG’s parameters across several data generating process (DGP) scenarios. While $\mu$ and $\sigma$ were set at 0 and 1 respectively (as no major impact of location and scale on the quality of the estimates was expected), $\tau$ and $\alpha$ were varied such that $\tau \in \{0.5,\, 1.25$} and $\alpha \in \{0.75,\, 1.75,\, 2.75\}$. Variations of these parameter values were assessed across small and large sample sizes such that $n\in \{50;\, 100;\, 200;\, 400;\, 800;\, 1600\}$. Each of the resulting 36 DGP was replicated 1000 times.

For each setting, the empirical bias, root mean squared error (rMSE), and coverage probability were estimated (see Table 2). The coverage probability had a 95% Wald confidence interval utilising the inverse of the observed Fisher information as the asymptotic covariance matrix for all four distributional parameters $\mu$, $\sigma$, $\tau$ and $\alpha$. The median value across the 1,000 simulations in each DGP was estimated for the bias and the rMSE. The empirical coverage probabilities were obtained by averaging over the replications. Given that the numerical estimation of MLE failed in the case of small sample sizes, the number of converged optimisations for each DGP is reported.

The results indicate that, as expected from standard asymptotic consistency and normality of MLEs, all criteria improve as the sample size increases, regardless of the specific parameter setting (i.e. the bias and the rMSE decrease while the coverage approaches the nominal level of 95%). It is evident too that larger values of either $\tau$ and/or $\alpha$ are associated with decreasing statistical performance; thus, larger sample sizes are required to obtain reliable estimates.

**Table 2:** Behaviour of the MLE for selected parameters of the GEG distribution

*n*	$\alpha$	$\tau$	$\sigma$	$\mu$	Converged	$\hat{\mu }$			$\hat{\sigma }$			$\hat{\tau }$			$\hat{\alpha }$		
						Bias	rMSE	Coverage	Bias	rMSE	Coverage	Bias	rMSE	Coverage	Bias	rMSE	Coverage
50	0.75	0.5	1	0	0.949	− 0.491	2.81	0.781	− 0.282	0.202	0.858	− 0.008	0.013	0.975	0.24	0.424	0.867
100	0.75	0.5	1	0	0.96	− 0.275	1.659	0.852	− 0.16	0.084	0.905	− 0.003	0.006	0.96	0.132	0.313	0.875
200	0.75	0.5	1	0	0.966	− 0.14	1.018	0.881	− 0.073	0.045	0.924	0.003	0.004	0.947	0.062	0.202	0.889
400	0.75	0.5	1	0	0.983	− 0.037	0.534	0.912	− 0.035	0.023	0.94	− 0.001	0.002	0.93	0.008	0.126	0.924
800	0.75	0.5	1	0	0.996	− 0.048	0.298	0.953	0	0.011	0.951	0.003	0.001	0.932	0.028	0.073	0.954
1600	0.75	0.5	1	0	1	0.006	0.164	0.946	0	0.006	0.951	0.002	0.001	0.951	0	0.043	0.945
50	1.75	0.5	1	0	0.968	0.032	1.042	0.825	− 0.444	0.355	0.876	− 0.018	0.007	0.968	− 0.087	1.445	0.905
100	1.75	0.5	1	0	0.976	− 0.104	1.108	0.873	− 0.275	0.137	0.895	− 0.014	0.004	0.957	0.068	1.384	0.923
200	1.75	0.5	1	0	0.986	− 0.09	0.866	0.842	− 0.169	0.057	0.91	− 0.006	0.002	0.941	0.108	1.144	0.879
400	1.75	0.5	1	0	0.997	0.003	0.689	0.838	− 0.11	0.025	0.933	− 0.004	0.001	0.947	0.008	0.836	0.832
800	1.75	0.5	1	0	1	− 0.034	0.296	0.905	− 0.042	0.009	0.948	0.001	0.001	0.953	0.042	0.402	0.91
1600	1.75	0.5	1	0	1	− 0.026	0.192	0.93	− 0.025	0.005	0.948	− 0.001	0	0.928	0.016	0.256	0.936
50	2.75	0.5	1	0	0.972	0.433	1.122	0.741	− 0.456	0.476	0.888	− 0.034	0.008	0.966	− 0.772	3.231	0.834
100	2.75	0.5	1	0	0.977	0.195	0.762	0.861	− 0.339	0.183	0.926	− 0.017	0.003	0.971	− 0.361	2.443	0.9
200	2.75	0.5	1	0	0.986	0.062	0.642	0.921	− 0.202	0.079	0.953	− 0.007	0.002	0.96	− 0.004	2.122	0.938
400	2.75	0.5	1	0	1	0.014	0.531	0.908	− 0.132	0.034	0.943	− 0.003	0.001	0.948	0.045	1.86	0.925
800	2.75	0.5	1	0	1	− 0.026	0.354	0.877	− 0.064	0.012	0.967	− 0.001	0	0.963	0.062	1.101	0.855
1600	2.75	0.5	1	0	1	− 0.012	0.21	0.902	− 0.037	0.006	0.964	0	0	0.938	0.034	0.701	0.907
50	0.75	1.25	1	0	0.942	0.266	2.868	0.817	− 0.035	0.364	0.9	2.304	5.307	0.95	0.044	0.516	0.907
100	0.75	1.25	1	0	0.958	0.046	2.029	0.855	0.111	0.304	0.79	0.775	0.69	0.987	0.204	0.481	0.909
200	0.75	1.25	1	0	0.974	− 0.179	1.405	0.875	0.16	0.234	0.699	0.122	0.183	0.986	0.336	0.45	0.875
400	0.75	1.25	1	0	0.995	− 0.184	1.055	0.873	0.11	0.173	0.743	0.077	0.065	0.99	0.238	0.365	0.789
800	0.75	1.25	1	0	0.999	− 0.093	0.526	0.888	0.035	0.068	0.853	0.022	0.023	0.985	0.083	0.216	0.857
1600	0.75	1.25	1	0	1	− 0.072	0.364	0.875	0.026	0.047	0.878	0.01	0.013	0.976	0.062	0.167	0.884
50	1.75	1.25	1	0	0.939	0.154	3.068	0.797	− 0.261	0.41	0.874	− 0.077	0.327	0.942	0.002	2.818	0.876
100	1.75	1.25	1	0	0.892	0.145	2.464	0.833	− 0.115	0.302	0.837	− 0.025	0.146	0.958	0.117	2.676	0.897
200	1.75	1.25	1	0	0.913	0.024	2.098	0.838	− 0.004	0.244	0.694	0.009	0.08	0.965	0.071	2.554	0.88
400	1.75	1.25	1	0	0.933	− 0.109	1.506	0.872	0.045	0.168	0.73	0.02	0.033	0.988	0.255	2.218	0.801
800	1.75	1.25	1	0	0.964	0.026	0.896	0.854	− 0.008	0.085	0.853	− 0.001	0.014	0.982	− 0.026	1.532	0.868
1600	1.75	1.25	1	0	0.994	− 0.008	0.468	0.892	0.012	0.046	0.891	0.007	0.006	0.991	0.042	1.022	0.909
50	2.75	1.25	1	0	0.928	0.442	2.564	0.761	− 0.437	0.408	0.847	− 0.13	0.202	0.969	− 0.768	6.623	0.846
100	2.75	1.25	1	0	0.904	0.264	2.554	0.783	− 0.246	0.322	0.807	− 0.102	0.103	0.954	− 0.177	6.685	0.847
200	2.75	1.25	1	0	0.855	0.236	2.189	0.835	− 0.143	0.219	0.734	− 0.039	0.051	0.951	− 0.527	6.158	0.885
400	2.75	1.25	1	0	0.869	0.27	1.721	0.831	− 0.094	0.15	0.761	− 0.019	0.026	0.969	− 0.581	5.229	0.849
800	2.75	1.25	1	0	0.924	0.152	1.056	0.868	− 0.038	0.086	0.859	− 0.01	0.011	0.978	− 0.327	3.851	0.872
1600	2.75	1.25	1	0	0.963	0.072	0.678	0.907	− 0.039	0.057	0.898	− 0.006	0.004	0.985	− 0.205	2.825	0.922

## Illustration of the generalised exponential-Gaussian distribution via published data sets

In this section, the versatility of the GEG distribution is illustrated via four data sets in which motor or neuronal RTs are featured.

- *ADHD’s simple RTs in *Osmon et al. (2018) *[here ADHD data set]*. Osmon et al. (2018) obtained three different types of RTs from 27 neurotypical participants and 28 participants diagnosed with attention-deficit/hyperactivity disorder (ADHD). The RTs obtained during the simple RT task are featured in this study. In this task, participants had to press the same centrally located key when a stimulus appeared, regardless of the location on a computer screen (right or left side). As each participant had a fixed number of 120 trials and there were 28 ADHD participants, a total of 3360 trials were obtained.
- *Monkey S’s RTs in a reaching task in *Kuang et al. (2016) *[here M.S. data set]*. The goal of this study was to investigate the neuronal activity in the posterior parietal cortex in two rhesus monkeys while they performed centre-out hand reaches under either normal or prism-reversed viewing conditions. All the trials in all 107 sessions for the ‘normal right’ condition from monkey S were used. There were between 19 and 62 trials across sessions and the median number of trials across sessions was $40 \pm 14.82$. This gives a total of 3996 trials and RTs. After removing RTs < 50 ms, 3980 RTs remained. It is important to acknowledge that the lower limit of 50 ms is somewhat arbitrary and there are no agreed rules in the monkey literature on what the lower and upper boundaries should be. However, considering usual visual information processing latencies (ventral stream: $\sim$50 ms; dorsal stream: $\sim$100 ms), imposing a 50 ms constraint seems to be a minimal requirement.
- *Crows’ RTs in a visual task in *Veit et al. (2014) *[here C.D. data set]*. This study aimed to investigate the neuronal correlates of visual working memory in four trained carrion crows. The experimental set-up required crows to remember a visual stimulus for later comparison while the activity of neurons in the nidopallium caudolaterale (a higher association brain area functionally akin to the prefrontal cortex in monkeys) was recorded. Veit et al. (2014) reported a histogram of the RTs of 162 visually responsive neurons (i.e. neurons from the four crows for which RTs could be estimated) in Figure 4B in their paper.
- *Synchronised cortical state and neuronal RTs [here S.S.N. data set]*. Fazlali et al. (2016) investigated the link between spontaneous activity in the locus coeruleus (a key neuromodulatory nucleus in the brainstem) and synchronised/desynchronised states in the vibrissal barrel somatosensory cortex (BC) in Wistar rats. One of the analyses looked at neuronal responses in the BC during the two cortical states. The authors found that neuronal RTs in the BC were faster during the desynchronised than during the synchronised cortical state (in their study neuronal RTs were defined as the first time bin exceeding background activity by three standard deviations). The distribution of the BC’s neuronal RTs in the synchronised cortical state are featured in this study. Figure 6B and the section ‘Reduced response latency in desynchronised state’ in Fazlali et al. (2016) provide details for this data set.

Table 3 reports the goodness-of-fit of the GEG and other distributions to these data sets (to improve numerical stability, all data were divided by a constant factor of 100). The results indicated that the SNAP and SN distributions provided the best fits in the C.D. and S.S.N. data sets, respectively, and that the GEG distribution gave the best fit in the remaining two data sets (bearing in mind that the lower the AIC and/or BIC, the better the fit). Note that, while we are comparing distributions with differing numbers of parameters and therefore an inherent advantage for more complex distributions to fit the data better, AIC and/or BIC both adjust for the model complexity such that we can make valid comparisons across distributions with different numbers of parameters.

Although the ECSN (Exponential-Centred Skew-Normal) distribution gave the second-best fit in the C.D. data set, this result is not reliable given difficulties in convergence, leaving that second place for the GEG distribution. In the case of the S.S.N. data set, the LN and GEG distributions gave the second- and third-best fits, respectively.

**Fig. 2:**
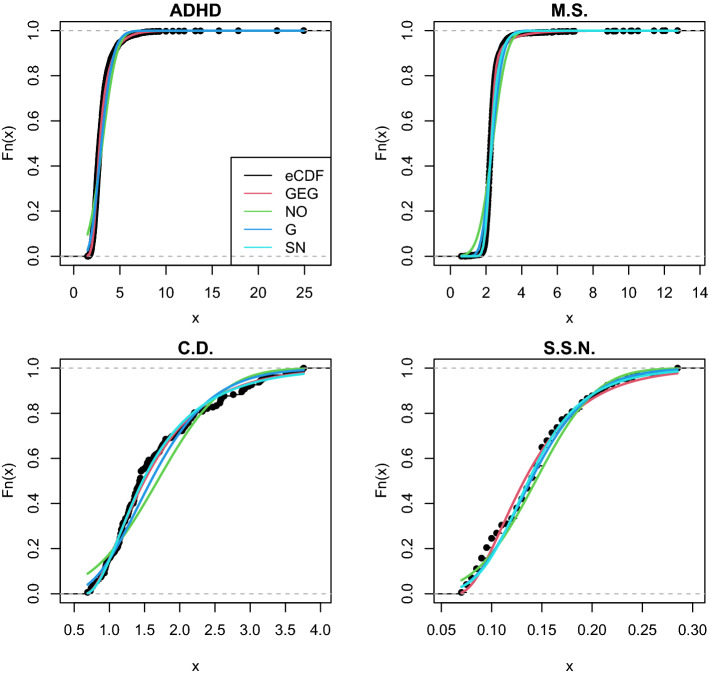
Empirical CDFs of four real-life data sets and four fitted theoretical CDFs. The data distributions are represented by black dots (eCDF). Note that the NO distribution tends to miss the tails of the data (e.g. in data sets M.S. and C.D.) and in other cases it misses the data locations (e.g. in the C.D. data set). Note there is a trade-off between interpretability and fitness (i.e. accuracy and flexibility) that requires careful consideration when selecting a distribution to model data. GEG = four-parameters Generalised Exponential-Gaussian distribution; NO = two-parameter Normal distribution; G = two-parameter Gamma distribution; SN = three-parameter Skew-Normal distribution. The *x* axis represents RTs (these were divided by 100 to improve numerical stability). See Table 3 for the results of the fits

Table 3 also shows that the NO distribution tended to give bad fits (i.e. very high AIC and/or BIC) due to its natural inability to fit asymmetric data and its definition on the real line, which does not match with the non-negativity of RTs. Such a result reinforces the claim that methods that assume normality in the response variable (e.g. ordinary least squares, *t*-test and ANOVA) are not suitable to analyse and model RT data. The results of the LN distribution also indicate that a logarithmic transformation is usually not enough to make the distribution of RTs adhere to a normal law. That the EXP distribution gave the highest AIC and/or BIC suggests that having just one parameter (rate or inverse scale in the case of this distribution) limits this distribution’s flexibility to meet the shape of RT data. Overall, the results thus suggest the GEG distribution provides a good fit to these behavioural and neuronal RTs data sets (see Fig. 2). A future study should aim to fit several suitable distributions to a much larger collection of real-life neuronal and behavioural data sets to obtain a fine-grained picture of which distributions tend to give the best fit across data sets of comparable characteristics.

**Table 3:** Parameter and goodness-of-fit estimates of 10 distributions fitted to four data sets

Dataset (size)	Distribution	Parameter (SE)				AIC|BIC
		$\mu$	$\sigma$	$\tau$	$\alpha$	
ADHD (*n* = 3360)	EXP	3.105 (0.054)	NA (NA)	NA (NA)	NA (NA)	14335.086|14341.205
	NO	3.105 (0.021)	1.231 (0.015)	NA (NA)	NA (NA)	10936.058|10948.298
	T	2.831 (0.013)	0.584 (0.011)	2.449 (0.096)	NA (NA)	8872.626|8890.985
	EG	2.109 (0.010)	0.264 (0.010)	0.996 (0.015)	NA (NA)	8215.94|8234.30
	G	3.105 (0.017)	0.316 (0.004)	NA (NA)	NA (NA)	9171.67|9183.91
	LN	2.951 (0.015)	0.297 (0.004)	NA (NA)	NA (NA)	8652.98|8665.22
	SNAP	3.061 (0.043)	2.943 (0.008)	15.521 (0.913)	0.120 (0.081)	8379.29|8403.77
	SN	2.02 (0.009)	0.516 (0.002)	3.321 (0.012)	NA (NA)	9077.44v9095.80
	ECSN	1.856 (0.056)	0.387 (0.054)	1.015 (0.024)	1.634 (0.611)	8215.42|8239.90
	**GEG**	**2.471 (0.078)**	**0.253 (0.009)**	**0.835 (0.044)**	**0.486 (0.074)**	**8197.86|8222.34**
M.S (*n* = 3980)	EXP	2.371 (0.038)	NA (NA)	NA (NA)	NA (NA)	14832.983|14839.272
	NO	2.371 (0.01)	0.631 (0.007)	NA (NA)	NA (NA)	7632.219|7644.798
	T	2.263 (0.004)	0.198 (0.003)	2.029 (0.066)	NA (NA)	2650.054|2668.921
	EG	2.012 (0.005)	0.191 (0.002)	0.358 (0.003)	NA (NA)	3290.43|3309.29
	G	2.371 (0.008)	0.201 (0.002)	NA (NA)	NA (NA)	5273.32|5285.89
	LN	2.323 (0.007)	0.183 (0.002)	NA (NA)	NA (NA)	4499.72|4512.30
	SNAP	− 17.444 (NaN)	2.694 (0.004)	37.56 (0.021)	3104014918865.67 (NaN)	4170.55|4195.70
	SN	2.053 (0.006)	0.398 (0.001)	1.974 (0.004)	NA (NA)	5669.91|5688.78
	ECSN	2.013 (NaN)	0.191 (0.004)	2.79 (0.059)	− 0.002 (NaN)	3292.42|3317.58
	**GEG**	**2.503 (0.011)**	**0.071 (0.002)**	**0.807 (0.065)**	**0.061 (0.005)**	**2546.67|2571.83**
C.D. (*n* = 162)	EXP	1.684 (0.132)	NA (NA)	NA (NA)	NA (NA)	494.874|497.962
	NO	1.684 (0.058)	0.737 (0.041)	NA (NA)	NA (NA)	364.894|371.069
	T	1.6 (0.056)	0.644 (0.042)	7.818 (3.184)	NA (NA)	365.753|375.016
	EG	0.901 (0.031)	0.164 (0.040)	0.783 (0.069)	NA (NA)	309.08|318.34
	G	1.684 (0.054)	0.411 (0.022)	NA (NA)	NA (NA)	325.43|331.61
	LN	1.544 (0.050)	0.410 (0.023)	NA (NA)	NA (NA)	315.55|321.72
	**SNAP**	**1.102 (0.133)**	**1.282 (0.038)**	**8.929 (1.792)**	**0.459 (1.653)**	**306.89|319.24**
	SN	0.893 (0.028)	0.350 (0.019)	3.235 (0.165)	NA (NA)	310.58|319.84
	ECSN	0.651 (0.028)	0.331 (0.080)	1.302 (0.136)	354.056 (11863.415)	308.53|320.88
	GEG	0.663 (0.038)	0.003 (4.123)	1.447 (0.143)	1.946 (0.361)	308.76|321.11
S.S.N. (*n* = 171)	EXP	0.144 (0.011)	NA (NA)	NA (NA)	NA (NA)	− 319.179|− 316.038
	NO	0.144 (0.004)	0.047 (0.003)	NA (NA)	NA (NA)	− 553.518|− 547.234
	T	0.143 (0.004)	0.046 (0.003)	27.098 (31.564)	NA (NA)	− 551.764|− 542.339
	EG	0.102 (0.003)	0.027 (0.005)	0.042 (0.004)	NA (NA)	− 565.531|− 556.106
	G	0.144 (0.004)	0.324 (0.017)	NA (NA)	NA (NA)	− 572.003|− 565.719
	LN	0.136 (0.003)	0.327 (0.018)	NA (NA)	NA (NA)	− 573.931|− 567.648
	SNAP	− 1.166 (1.508)	0.686 (0.353)	− 2.818 (3.024)	2162326935.754 (43972887567.382)	− 568.638|− 556.071
	**SN**	**0.085 (0.002)**	**0.025 (0.002)**	**3.162 (0.178)**	NA (NA)	**− 580.824|− 571.399**
	ECSN	0.103 (0.128)	0.028 (0.004)	24.38 (3.841)	− 0.006 (5.82)	− 563.553|− 550.986
	GEG	0.063 (0.008)	0.002 (0.035)	21.475 (2.345)	2.716 (1.041)	− 572.157|− 559.59

The number of parameters of each distribution is shown in brackets. EXP = Exponential (1), NO = Normal (2), T = *t*-distribution (3), EG = Ex-Gaussian (3), G = Gamma (2), LN = Log-Normal (2), SNAP = Skew-Normal Alpha-Power (4), SN = Skew-Normal (3), ECSN = Exponential-Centred Skew-Normal (4), GEG = Generalised Exponential Gaussian (4). The sample size of each data set (*n*) is shown in brackets. ADHD = simple RTs of participants with ADHD (RTs correspond to several trials from several ADHD participants; no RTs discarded), M.S = reaching RTs of monkey S to a visual target presented on the right side under a normal viewing condition (RTs are from several trials across several sessions; RTs > 50 ms were retained), C.D = neuronal RTs of an adult carrion crow during a visual task, and S.S.N. = response latencies of synchronised state neurons (synchronised state was defined as oscillations of high amplitude and low frequency (<4 Hz)). Lowest AIC|BIC estimates are shown in bold. SE = standard error. Note the SNAP distribution gave biased estimates in the M.S. and S.S.N. data sets, and the ECSN distribution gave biased estimates in the M.S., C.D., and S.S.N. data sets. These situations reflect difficulties with convergence in numerical optimisation of the MLEs

## GAMLSS in a nutshell: a regression framework for distributional modelling

**Fig. 3:**
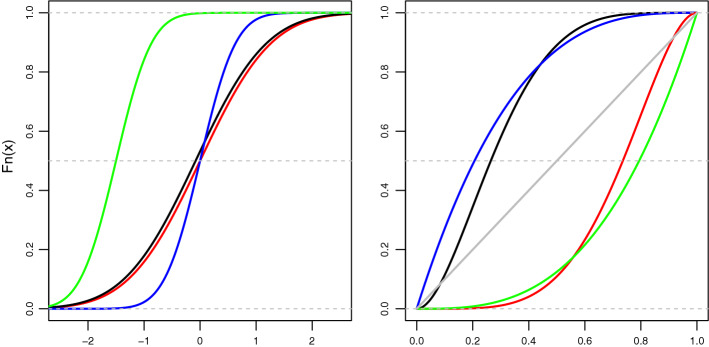
Cumulative distribution functions (CDF) illustrating different normal (left plot) and non-normal distributions (right plot). Left plot (differences/similarities in location and scale): black and red CDFs have similar location and similar scale; blue and black/red CDFs have similar location and different scale; blue and green CDFs have different location and similar scale; green and black/red CDFs have different location and different scale. In all these cases, LM and GLM would be able to identify similarities/differences in location only (i.e. mean values); that is, standard techniques are good for detecting shifts but not shapes of distributions. Right plot (different types of shapes): black and blue CDFs represent distributions with positive skew, green and red CDFs represent distributions with negative skew, and the grey CDF represents a uniform distribution. The dotted grey horizontal line cuts through the distributions medians

In the regression context, it is traditional to investigate the effects of independent variables (IVs) on the mean of the dependent variable (DV) and this is achieved via ordinary least squares regression (also known as linear models, LM). Improvements on the LM approach have been reflected in the generalised linear model (GLM) by replacing the required normal distribution of the response variable with the exponential family of distributions (e.g. the Gamma distribution). Although GLM is more flexible than LM, both focus on the effects of the IVs on the DV’s mean. However, even if LM and GLM could also model the effects of the IVs on the DV’s standard deviation, the findings would be limited to the data’s location and scale parameters. Additionally, LM allows only a linear relationship between continuous IVs and DVs, and GLM assumes a linear relationship between the transformed response in terms of the link function and IVs. Generalised additive models (GAM), however, allow modelling such a relationship by using non-parametric (smooth) functions on the numeric IVs. Generalised additive models for location, scale and shape (GAMLSS) is the only existing regression framework that encompasses all these regression methods (Stasinopoulos et al. 2018; Kneib et al. 2021). It also has the extra property of allowing modelling of the effects of IVs on the DV’s location, scale and shape (i.e. skewness and kurtosis) via over 100 statistical distributions, thus enabling a comparison between many different models and proper distributional analysis [Rigby et al. (2020); see also Fig. 3].

Statistically speaking, in a GAMLSS model $Y_i \sim D(\varvec{\theta })$ where the values of $Y_i$ are *n* independent observations, for $i=1,2,\ldots ,n$, and that have probability (density) function $f_Y(y_i|{\varvec{\theta }})$ conditional to distribution parameters, usually up to four distribution parameters, each of which can be a function of the IVs. For $k=1,2,3,4$, let $g_k(.)$ be a known monotonic link function relating a distribution parameter to a predictor $\varvec{\eta }_k$, such that

$$\begin{aligned} \begin{aligned} g_k(\varvec{\theta })=\varvec{\eta }_k={\mathbf{X}}_k \varvec{\beta }_k+\sum _{j=1}^{J_k}s_{kj}({\mathbf{x}}_{kj}) \end{aligned} \end{aligned}$$

where $\mathbf{X}_k$ is a known design matrix, ${\beta }_k=(\beta _{k1}, \ldots , \beta _{k J_{k}^{'}} )^{\top }$ is a parameter vector of length $J_{k}^{'}$, $s_{kj}$ is a smooth non-parametric function of variable $X_{kj}$ and the terms $\mathbf{x}_{kj}$ are vectors of length *n*, for $k=1,2,3,4$ and $j=1,\ldots ,J_k$. Here, $\varvec{\theta }$ $=(\tau , \mu , \sigma , \alpha )$, *D* represents the GEG distribution, and each of the GEG distribution parameters can be modelled as linear or smooth functions of the IVs.

In a nutshell, GAMLSS is thus an interpretable, flexible and sophisticated framework for data modelling. It is interpretable in that it is conceived in the well-known regression framework; it is flexible in that it allows modelling of the response variable via several candidate statistical distributions; and it is sophisticated in that numeric and categorical IVs can be subjected to cutting-edge smoothing algorithms. Thus, GAMLSS could be an educated analytical approach to respond to the current lack of statistical sophistication and rigour permeating research in neuroscience (Nieuwenhuis et al. 2011).

To illustrate the potential of GAMLSS for the analysis of RT data, the data set shown in Figure 2F in Schledde et al. (2017) was examined via GAMLSS. In that study, the authors recorded in monkey motion-sensitive area MT and investigated the latency of neurons in response to stimulus changes under different conditions of attention. Two monkeys were engaged in a change-detection paradigm and the neuron under investigation was responding to a speed change either with spatial attention directed to its receptive field or away from it (i.e. attend-in and attend-out), and with feature-attention directed towards the motion domain or towards the colour domain otherwise (i.e. speed and colour tasks). The authors found that the response latency of the neurons was significant, depending on the attentional condition, with both spatial and feature attention having an influence on the shape of the distribution of latencies.

Such data can be represented as the regression model ‘$RT \sim A * T$’; where RT are the neuronal latencies, *A* and *T* are the 2-level categorical variables attention (attend-in and attend-out) and task (colour and speed), respectively, and ‘$*$’ stands for main effects and interactions. That model is equivalent to a $2\times 2$ ANOVA design and that is traditionally assessed via LM. GAMLSS models therefore imply four conditional distributions whose ECDFs are shown in Figure 2F in Schledde et al. (2017). There can be several analytical options, but for illustration purposes only a main-effects-with-no-random-effects model was considered. A GAMLSS modelling of the conditional distributions via the 10 probability distributions considered in Table 3 indicated that while the G distribution gave the best fit for one conditional distribution, the NO distribution fitted the three remaining conditional distributions best. A marginal distributional modelling (i.e. all the RTs) showed the EG distribution gave the best fit (the GEG distribution being the second-best fit). Two versions of the regression model shown above were considered; a model in which the DV was modelled via the NO distribution and a model in which the DV was modelled via the EG distribution.^3^ While the NO model corresponds to the classic LM, the EG model is achievable only via GAMLSS. In both cases only the location parameter was investigated and RTs were divided by 100 to improve numerical stability. The results showed that the EG model (AIC = − 73.86) provided a better fit to the data than the NO model (AIC = − 70.09).^4^

## Discussion and conclusion

The GEG distribution was proposed as a candidate statistical model of behavioural and neuronal RTs and its statistical properties were described and examined via simulations. Given that the GEG is a four-parameter distribution, it can readily adopt non-normal shapes typically found in RT data; and this was exemplified via real-life data sets. It is a common practice to apply non-linear transformations to RT data to meet parametric assumptions and thus approximate normality or improve symmetry. However, the GEG distribution, and other distributions considered here enable working with the original shape of the data and therefore sidestep unnecessary non-linear transformations. The following paragraphs discuss statistical and practical implications of the GEG distribution within a GAMLSS framework for the analysis of neuronal and behavioural RTs.

### Some statistical graphics aspects relating to GAMLSS

Commenges and Seal (1986) argue that explaining the relationship between neuronal RTs and behavioural RTs in well-controlled experiments, depends on the statistical methods used for the data analysis. The GEG distribution is amenable to properly characterise the distribution of both types of RTs conditioned on the specific variables manipulated in an experiment. However, the explanatory power of the GEG can only be appreciated when this distribution is used within a distributional modelling approach. Such a method was briefly described above: GAMLSS (Stasinopoulos et al. 2017). A key step, though, in the distributional modelling of data is the use of statistical graphical techniques that allow the distribution of the data to be examined. Traditionally, bar plots have been used for such a purpose but they do not allow the shape of the data to be visualised; instead, boxplots and violin plots are better techniques. However, empirical cumulative distribution function (ECDF) plots are the optimal approach to investigate the shape of data and are instrumental in comparing vectors of data. An example of ECDFs representing neuronal RTs can be seen in Figure 4A in Mormann et al. (2008). Indeed, Mormann et al. (2008)’s study is an excellent example of how neuronal RTs can provide insight as to neuronal firing associated to brain connectivity and stimuli.

### A hypothetical scenario where GAMLSS can be used

The explanatory and predictive power of statistical distributions can only be achieved via the GAMLSS framework. The following lines depict a hypothetical experiment in which GAMLSS could be used to model RTs via the GEG distribution to better understand neuronal activity. A study conceptualised from the the two-stream model of higher-order visual processing (Goodale and Milner 1992) measures extracellular RTs in neurons specialised in the shape of visual stimuli. The study’s goal is to characterise neuronal RTs conditioned on IVs of interest in the experiment; of particular interest is the presentation time of the stimuli. Hence, single- and multiple-neuron recordings are performed in cortical visual areas V1, V2, V3, and V4 from a small sample of neurotypical human adult participants. The researchers define neuronal RTs as the time lapse between the presentation of the stimulus and the moment the neuron generates an action potential. Since each neuron ‘sees’ all stimuli, RT distributions per neuron for all trials and stimuli are obtained. Further suppose that data from a reasonable number of neurons in each V area are obtained (e.g. 10 neurons per area per participant). The stimulus consists of equal numbers of two-dimensional round and angular shapes of equal size and colour (e.g. all black colour). The task consists of sacadding from a fixation point to selected coordinates on a computer screen where the shapes are shown individually and randomly at a fixed interstimulus interval but at three different presentation times (e.g. stimuli are exposed for 10, 30, and 50 ms; i.e. each image is seen three times in total).

A traditional LM or GLM model to analyse the data could be conceived as $RT \sim V * T * S$, such that *RT*, *V*, *T*, and *S* stand for the resulting marginal distribution of neuronal RTs, the four V cortical areas, the three presentation times, and the two types of shapes, respectively; and $*$ stands for the main effects and interactions. The results would inform whether, for example, there are differences in mean RTs among the four V areas, the three presentation times, and/or the two types of shapes. Also, the model would indicate, for example, if there is an interaction between *V* and *T* such that differences in mean RTs between V areas may occur at certain presentation times. The other two two-way interactions and the three-way interaction could also be investigated. The issue with modelling such data via LM or GLM is that the findings are limited to mean RTs. Regardless of the main or interaction effects on the mean RTs, it may be the case that there are effects on the RTs’ variability (i.e. the RT’s scale). Neither LM nor GLM can detect potential effects of the IVs on changes in the RT’s scale. GAMLSS, on the other hand, is able to examine effects that the IVs can have on the RT’s location parameter (as LM and GLM do) but can also determine if the same IVs (or a subset of them) can affect the response’s variability (see Fig. 3). Importantly, while LM can model the DV via the Normal distribution only and GLM can do so via distributions from the Exponential family, GAMLSS can use those and any other statistical distribution implemented in the gamlss.dist R package (Rigby et al. 2020) or in a way that can be used within the GAMLSS framework [see Roquim et al. (2021) and the ‘RelDists’ package for examples of this approach].

Modelling the data of this hypothetical experiment via GAMLSS would allow understanding of the trial-to-trial RT signature of single and multiple neurons conditioned on the stimuli and task they are faced with. Furthermore, it could be inferred that signature RTs’ distributions (location, scale and shape) should be able to differentiate between healthy and unhealthy neurons according to task demands (e.g. visual tasks, auditory tasks, type of stimuli). If there is a predictive goal, trees and forest for distributional regression could be used [see Schlosser et al. (2019) and the R package disttree], since they blend algorithmic modelling with GAMLSS modelling.

### Applied implications of the GAMLSS modelling

The previous example illustrates how neuronal RTs can be used to explain the processing of stimuli in networks of brain areas. An actual example of the value of investigating neuronal RTs is provided in Figures 5D and 5E in Yoshor et al. (2007). Those figures show an increasing trend in neuronal RTs (Figure 5D) and time to peak (Figure 5E) from zone 1 (areas for shape and colour processing) to zone 4 (areas for object and face processing) in the visual cortex; in particular, there was an RT mean difference between neurons in zone 1 and zone 4. In a similar vein, Mormann et al. (2008) found that parahippocampal, entorhinal, hippocampal, and amygdalan neuronal average RTs were in accord with neuroanatomical evidence. Also, Lin et al. (2018) found increments in RTs’ average and variability from the calcarine fissure (low-order primary sensory cortex) to the fusiform gyrus (higher-order association area), areas within the ventral pathway of face processing. Such types of analytical approach contribute to better characterising brain areas and their functions by placing neurons along a sensory-motor spectrum (DiCarlo and Maunsell 2005). Very likely, a more sophisticated analysis of this type of data via GAMLSS would further the current understanding of neuronal activation in those areas.

### Future challenges

Although using the GEG distribution for the analysis of RTs offers a promising avenue for new research, there are some aspects that need to be investigated. It was argued that the GEG is a four-parameter distribution flexible enough to capture changes in location, scale, and shape. The simulation study reported above indicated that the GEG’s scale parameters, that is, skewness and kurtosis, require large samples to allow reliable estimates. Future work should investigate these parameters in more depth. A way to do so is via transformed moment kurtosis and skewness plots [see section 16.1.2 in Rigby et al. (2020)]. These types of plot enable the investigation of regions of possible combinations of transformed moment skewness and transformed kurtosis of the distributions, and so the flexibility of the GEG can be compared to other continuous distributions in terms of moment skewness and kurtosis.

### Conclusion

In summary, distributional modelling of neuronal RTs enables fine-grained temporal profiles of brain areas and networks. The GEG has been proposed as a suitable distribution to fit RT data and its use in neuronal and behavioural statistical modelling will contribute to forging the link between neurometrics and psychometrics. Data sets featured in this article and related R codes are available at https://cutt.ly/IWJeSkO.

## Appendix

### Statistical details of the Ex-Gaussian distribution

For $Y_1\sim AP(\tau )$ and $Y_2\sim N(\mu ,\sigma ^{2})$ with independent $Y_1$ and $Y_2$, it follows that the PDF of the random variable $X=Y_1+Y_2$ is given by:

8 $$\begin{aligned} f_{EG}(x)&= \frac{1}{\tau }e^{-\frac{x-\mu }{\tau }+\frac{\sigma ^{2}}{2\tau }}\Phi \left( \frac{x-\mu }{\sigma } -\frac{\sigma }{\tau }\right) ,\nonumber \\&\quad \tau>0,~~-\infty<\mu <\infty ,\quad \sigma >0. \end{aligned}$$

This PDF is denoted as $EG(\tau ,\mu ,\sigma ^{2})$. On the other hand, the EG distribution’s CDF can be obtained as:

9 $$\begin{aligned} F_{EG}(x)&= \int _{-\infty }^x\frac{1}{\tau }e^{-\frac{t-\mu }{\tau }+\frac{\sigma ^2}{2\tau ^2}}\Phi \left( \frac{t-\mu }{\sigma }-\frac{\sigma }{\tau }\right) dt \nonumber \\&= \frac{\sigma }{\tau }e^{\frac{\sigma ^2}{2\tau ^2}}\int _{-\infty }^{\frac{x-\mu }{\sigma }-\frac{\sigma }{\tau }}e^{-\frac{\sigma }{\tau }\left( v+ \frac{\sigma }{\tau }\right) }\Phi \left( v\right) dv \nonumber \\&= \frac{\sigma }{\tau }e^{-\frac{\sigma ^2}{2\tau ^2}} \int _{-\infty }^{\frac{x-\mu }{\sigma }-\frac{\sigma }{\tau }}e^{-\frac{\sigma }{\tau }v}\Phi \left( v\right) dv \nonumber \\ &= e^{-\frac{\sigma ^2}{2\tau ^2}}\left[ -e^{-\frac{\sigma }{\tau }v}\Phi \left( v\right) +e^{\frac{\sigma ^2}{2\tau ^2}} \Phi \left( v+\frac{\sigma }{\tau }\right) \right] {\vert }_{-\infty }^{\frac{x-\mu }{\sigma }-\frac{\sigma }{\tau }}\nonumber \\&\quad \Phi \left( \frac{x-\mu }{\sigma }\right) -e^{-\frac{x-\mu }{\tau }+\frac{\sigma ^2}{2\tau ^2}}\Phi \left( \frac{x-\mu }{\sigma }-\frac{\sigma }{\tau }\right) \nonumber \\&= \Phi \left( z\right) -\tau f_{EG}(x), \end{aligned}$$

where $$z=\left( \frac{x-\mu }{\sigma }\right)$$.

From this result, it follows that the survival functions, relative risk and hazard, are given by:

10 $$\begin{aligned} S_{EG}(t)&= S_N(t)+\tau f_{EG}(t), \end{aligned}$$

11 $$\begin{aligned} r_{EG}(t)&= \frac{-e^{\frac{x-\mu }{\tau }+\frac{\sigma ^{2}}{2\tau }}\Phi \left( \frac{x-\mu }{\sigma }-\frac{\sigma }{\tau }\right) }{\tau \left( \Phi \left( \frac{x-\mu }{\sigma }\right) -e^{\frac{x-\mu }{\tau }+\frac{\sigma ^{2}}{2\tau }}\Phi \left( \frac{x-\mu }{\sigma }-\frac{\sigma }{\tau }\right) \right) },\nonumber \\ h_{EG}(t)&= \frac{e^{-\frac{x-\mu }{\tau }+\frac{\sigma ^{2}}{2\tau }}\Phi \left( \frac{x-\mu }{\sigma }-\frac{\sigma }{\tau }\right) }{\tau \left( S_N(t) +e^{-\frac{x-\mu }{\tau }+\frac{\sigma ^{2}}{2\tau }}\Phi \left( \frac{x-\mu }{\sigma }-\frac{\sigma }{\tau }\right) \right) }. \end{aligned}$$

where $S_N(t)$ is the survival function of the normal distribution.

Given the independence between the convoluted exponential and normal variables, it follows that

12 $$\begin{aligned} \mathbb{E}(X)=\tau +\mu ~~\text {and}\quad Var(X)=\tau ^{2}+\sigma ^{2}. \end{aligned}$$

Likewise, the skewness and kurtosis coefficients are given by:

13 $$\begin{aligned} skew=\frac{2\left( \frac{\tau }{\sigma }\right) ^{3}}{\left[ 1+ \left( \frac{\tau }{\sigma }\right) ^{2}\right] ^{3/2}}\quad \text {and} \quad kurt=\frac{3\left( 1+2\left( \frac{\tau }{\sigma }\right) ^{2}+3\left( \frac{\tau }{\sigma }\right) ^{4} \right) }{\left[ 1+ \left( \frac{\tau }{\sigma }\right) ^{2}\right] ^{2}}. \end{aligned}$$

A simplification of kurtosis expression could be through excess kurtosis, which is calculated as $3-kurt=3\frac{\left( \frac{\sigma }{\tau }\right) ^2-2}{\left( \left( \frac{\sigma }{\tau }\right) ^2+1 \right) ^2}.$

These formulas thus indicate that the EG distribution has positive asymmetry.

In the context of an RT experiment, if *T* is the RT or response time, then, for a random sample $T=(T_1, T_2,\ldots ,T_n)',$ with $T_i\sim {EG(\tau ,\mu ,\sigma )},$ the distribution of the statistics $T_{(n)}=max[T_1, T_2,\ldots ,T_n],$ where *max* denotes the maximum of the sample, and is given by

14 $$\begin{aligned} F_{T}(t)& P[T\le {t}]=P[T_1\le {t},T_2\le {t},\ldots ,T_n\le {t}]\nonumber \\&\quad = \prod _{i=1}^{n}F_{EG}(t_i)=\left\{ F_{EG}(t)\right\} ^n. \end{aligned}$$

Therefore, the PDF of *T* is given by the expression:

15 $$\begin{aligned} \varphi _{T}(t)&=n f_{EG}(t)\left\{ F_{EG}(t)\right\} ^{n-1} , \end{aligned}$$

which is an expression similar to the PDF of the random variable AP(n).

### Moments

Moments of the *r*th order statistics of the GEG distribution are not available in a closed-form expression, and thus need to be estimated numerically. Then, for $X\sim GEG(\tau , \mu , \sigma , \alpha )$ it follows that

16 $$\begin{aligned} \mu _r=\mathbb{E}(X^r)=\alpha \int _0^1u^{\alpha -1}F_{EG}^{-1}(u;\tau ,\mu ,\sigma )du \end{aligned}$$

where $F_{EG}^{-1}(\cdot ;\tau ,\mu ,\sigma )$ is the inverse function of the EG distribution with parameters $\tau ,\mu ,\sigma$. This distribution is available in R packages such as emg and gamlss.

The central moments, $\acute{\mu _r}=\mathbb{E}(X-\mathbb{E}(X))^r$, for $r=2,3,4$ can be calculated by the expressions,

$$\begin{aligned}&\acute{\mu _2}=\mu _2-\mu _1^2,~~\acute{\mu _3}=\mu _3-3\mu _2\mu _1+2\mu _1^3~~\text {and}~~\\&\qquad \acute{\mu _4}=\mu _4-4\mu _3\mu _1+6\mu _2\mu _1^2-3\mu _1^4. \end{aligned}$$

Also, the variance, coefficients of variation, skewness, and kurtosis are given as:

$$\begin{aligned}&Var(X)=\acute{\mu _2},~~ CV=\frac{\sqrt{Var(X)}}{\mu _1},~~\sqrt{\beta _1}=\frac{{\acute{\mu _3}}}{{[\acute{\mu _2}]^{3/2}}}~~\text {and}~~\\&\qquad \beta _2=\frac{{\acute{\mu _4}}}{{[\acute{\mu _2}]^{2}}}. \end{aligned}$$

Skewness and kurtosis coefficients in the range [− 1.20841, 2.01773] and [1.658303, 10.28841] were found when $\mu =0,~\sigma =1$, $\tau \in [0.2,200]$ and $\alpha \in [0.25,20]$. These intervals contain skewness and kurtosis ranges of the PN and EG distributions; thus indicating that the GEG distribution is more flexible in terms of skewness and kurtosis than the PN and EG distributions. Furthermore, the GEG distribution’s skewness and kurtosis ranges provide better coverage than those of the SN (Azzalini 1985), and the SNAP (Martínez-Flórez et al. 2014) distributions. These results also indicate that while the EG distribution can only fit positively skewed data, the GEG distribution can fit data with positive skew, negative skew, and data with kurtosis larger than that of the EG distribution.

### Log-likelihood function

The parameters of the $GEG(\tau ,\mu ,\sigma ,\alpha )$ distribution are estimated via a maximum likelihood method. Thus, for a random sample of size *n*,  $X_1, X_2,\ldots , X_n$, where for $i=1,2,\ldots ,n$, $X_i\sim GEG(\tau ,\mu ,\sigma ,\alpha )$; the log-likelihood function of the parameter vector $\theta =(\tau ,\mu ,\sigma ,\alpha )'$ is given by:

$$\begin{aligned} \ell (\theta )&= n\log (\alpha )+\sum _{i=1}^n\log (f_{EG}(x_i))\\&\quad +\,(\alpha -1)\sum _{i=1}^n\log \left[ \Phi \left( \frac{x_i-\mu }{\sigma }\right) -\tau f_{EG}(x_i)\right] . \end{aligned}$$

#### Score function

$U(\theta )=(U(\tau ),U(\mu ),U(\sigma ),U(\alpha ))',$ defined as the derivatives with respect to the parameters of the log-likelihood function are given by:

$$\begin{aligned} U(\tau )&= \frac{\partial \ell (\theta )}{\partial \tau }=\frac{1}{\tau }\sum _{i=1}^n\left( -1+\frac{x_i-\mu }{\tau }-\frac{\sigma ^2}{\tau ^2}\right. \\&\quad \left. + \frac{\sigma }{\tau }w_i\right) +(\alpha -1)\sum _{i=1}^n\frac{\left( -\frac{x_i-\mu }{\tau }-\frac{\sigma ^2}{\tau ^2}+ \frac{\sigma }{\tau }w_i\right) f_{EG}(x_i)}{F_{EG}(x_i)}, \\ U(\mu )&= \frac{\partial \ell (\theta )}{\partial \mu }=\frac{n}{\tau }-\frac{1}{\sigma }\sum _{i=1}^nw_i-(\alpha -1)\\&\quad \sum _{i=1}^n \frac{\frac{1}{\sigma }\phi \left( \frac{x_i-\mu }{\sigma }\right) -\tau \left( \frac{1}{\tau }-\frac{1}{\sigma }w_i\right) f_{EG}(x_i)}{F_{EG}(x_i)}, \\ U(\sigma )&= \frac{\partial \ell (\theta )}{\partial \sigma }=\frac{n\sigma }{\tau ^2}\sum _{i=1}^n \left( \frac{x_i-\mu }{\sigma ^2}+\frac{1}{\tau }\right) w_i-(\alpha -1)\\&\quad \sum _{i=1}^n\frac{\frac{x_i-\mu }{\sigma }\,\,\phi \left( \frac{x_i-\mu }{\sigma }\right) \left( \frac{\sigma }{\tau } -\frac{\tau }{\sigma }\left( \frac{x_i-\mu }{\sigma }+\frac{\sigma }{\tau }\right) w_i \right) f_{EG}(x_i)}{F_{EG}(x_i)} \end{aligned}$$

and

$$\begin{aligned} U(\alpha )=\frac{\partial \ell (\theta )}{\partial \alpha }=\frac{n}{\alpha }+\sum _{i=1}^n\log \left[ \phi \left( \frac{x_i-\mu }{\sigma }\right) - \tau f_{EG}(x_i)\right] \end{aligned}$$

where $w_i=\frac{\phi (z_i)}{\Phi (z_i)}$ with $z_i=\frac{x_i-\mu }{\sigma }$. The corresponding score equations are obtained by equating the above equations to zero. The score equations can be solved by iterative numerical methods and this, in turn, leads to maximum likelihood estimators.

#### Information matrices

The elements of the observed information matrix, $J(\theta ),$ defined as minus the second derivatives of the log-likelihood function with respect to the parameters denoted by $\kappa _{\tau \tau },~\kappa _{\mu \tau },~\kappa _{\sigma \tau },\cdots ,\kappa _{\alpha \alpha }$, are given by:

$$\begin{aligned} \kappa _{\tau \tau }&= -\sum _i \left( \frac{1}{\tau ^2}-\frac{2\sigma }{\tau ^2}z_i+\frac{2\sigma ^2}{\tau ^2}-\frac{\sigma }{\tau ^2}w_i-\frac{\sigma ^2}{\tau ^3}q_i\right) - \\&\quad (\alpha -1)\\&\quad \sum _i\left[ \frac{\left( \frac{\sigma }{\tau ^2}z_i+\frac{2\sigma ^2}{\tau ^2}-\frac{\sigma }{\tau ^2}w_i-\frac{\sigma ^2}{\tau ^2}q_i+\left( -\frac{\sigma }{\tau }z_i-\frac{\sigma ^2}{\tau ^2}+\frac{\sigma }{\tau }w_i\right) \left( -\frac{1}{\tau }+\frac{\sigma }{\tau ^2}z_i-\frac{\sigma ^2}{\tau ^3}+\frac{\sigma }{\tau ^2}w_i\right) f_{EG}(x_i)\right) }{F_{EG}(x_i)}\right. \\&\quad -\left. \frac{\left( -\sigma z_i-\frac{\sigma ^2}{\tau }+\sigma w_i\right) ^2f_{EG}^2(x_i)}{\tau ^2 F_{EG}^2(x_i)}\right] ,\\ \kappa _{\tau \mu }&= \frac{n}{\tau ^2}+\frac{1}{\sigma ^2}\sum _iq_i -(\alpha -1)\sum _i\left[ \frac{f_{EG}(x_i)\left[ \frac{1}{\sigma }\left( 1-\frac{\sigma ^2}{\tau }\right) w_i-\frac{1}{\tau }(\sigma z_i+q_i)+\frac{\sigma ^2}{\tau ^2}+1\right] }{F_{EG}(x_i)}+\right. \\&\quad \left. \frac{\frac{1}{\tau ^2}\left( \sigma z_i+\frac{\sigma ^2}{\tau }-\sigma w_i\right) ^2f^2_{EG}(x_i)}{F^2_{EG}(x_i)}\right] , \end{aligned}$$

$$\begin{aligned} \kappa _{\tau \sigma }&= \frac{2n\sigma }{\tau ^3}-\frac{n}{\tau ^2}\sum _i w_i- \sum _i\left( \frac{z_i}{\sigma }+\frac{1}{\tau }\right) \frac{\sigma }{\tau ^2}q_i+ \\&\quad (\alpha -1)\\&\quad \sum _i\left[ \frac{f_{EG}(x_i)\left[ \left( -\frac{\sigma }{\tau ^2}-\frac{z_iw_i}{\sigma }+\frac{1}{\tau }\left( z_i+\frac{\sigma }{\tau }\right) q_i\right) +\left( \frac{\sigma }{\tau }-\frac{\tau }{\sigma }\left( z_i+\frac{\sigma }{\tau }\right) w_i\right) \left( -\frac{1}{\tau }+\frac{\sigma }{\tau ^2}(z_i+w_i)-\frac{\sigma ^2}{\tau ^2}\right) \right] }{F_{EG}(x_i)}\right. \\&\quad +\left. \frac{f_{EG}(x_i)\left( \frac{1}{\sigma }z_i\phi (z_i)+f_{EG}(x_i)\left( \frac{\sigma }{\tau }-\frac{\tau }{\sigma }\left( z_i+\frac{\sigma }{\tau }\right) w_i\right) \right) \left( -\sigma z_i-\frac{\sigma ^2}{\tau }+\sigma w_i\right) }{\tau F_{EG}^2(x_i)}\right] ,\\ \kappa _{\tau \alpha } & =\frac{\sigma }{\tau }\sum _i\frac{\left( z_i+\frac{\sigma }{\tau }+w_i\right) f_{EG}(x_i)}{F_{EG}(x_i)}, \\ \kappa _{\mu \mu }&= \frac{1}{\sigma ^2}\sum _iq_i+\frac{(\alpha -1)}{\sigma ^2}\\&\quad \sum _i\left[ \frac{\phi (z_i)+\tau \sigma ^2f_{EG}(x_i)\left[ \left( \frac{1}{\tau }-\frac{1}{\sigma }w_i(\mu ,\sigma ,\tau )\right) ^2- \frac{1}{\sigma ^2}q_i\right] }{F_{EG}(x_i)}\right. +\\&\quad \left. \frac{\left( \frac{1}{\sigma }\phi (z_i)+\tau f_{EG}(x_i)\left( \frac{1}{\tau }-\frac{1}{\sigma }w_i(\mu ,\sigma ,\tau )\right) \right) ^2}{F_{EG}(x_i)}\right] ,\\ \kappa _{\mu \sigma }&= -\frac{1}{\sigma ^2}\sum _iw_i(\mu ,\sigma ,\tau )+\frac{1}{\sigma ^2}\sum _iz_iq_i+ \frac{1}{\sigma \tau }\sum _iq_i- \\&\quad \frac{(\alpha -1)}{\sigma ^2}\\&\quad \sum _i\left[ \frac{\phi (z_i)(1-z_i^2)-\tau \sigma ^2f_{EG}(x_i)\left[ \left( \frac{\sigma }{\tau ^2}- \frac{1}{\sigma }\left( z_i+\frac{\sigma }{\tau }\right) w_i\right) \left( \frac{1}{\tau }\frac{1}{\sigma }w_i\right) \right] }{F_{EG}(x_i)}\right. \\&\quad +\left. \frac{\left( \sigma z_i\phi (z_i)+\sigma ^2\tau f_{EG}(x_i)\left[ \frac{\sigma }{\tau ^2}- \frac{1}{\sigma }\left( z_i+\frac{1}{\tau }\right) w_i\right] \right) ^2}{F^2_{EG}(x_i)}\right] ,\\ \kappa _{\mu \alpha }&= \frac{1}{\sigma }\\&\quad \sum _i\frac{\phi (z_i)}{F_{EG}(x_i)}+ \sum _i\frac{\left( 1-\frac{\tau }{\sigma }w_i(\mu ,\sigma ,\tau )\right) }{F_{EG}(x_i)}, \\ \kappa _{\sigma \sigma }&= -\frac{n}{\tau ^2}+\sum _i\left( -\frac{2}{\sigma ^2}z_iw_i+\left( \frac{1}{\sigma }z_i+\frac{1}{\tau }\right) ^2q_i\right) + \\&\quad (\alpha -1)\\&\quad \sum _i\left[ \frac{\frac{1}{\sigma ^2}\left( -2+z_i^2\right) z_i\phi (z_i)+f_{EG}(x_i)\left( \frac{1}{\tau }+\frac{2\tau }{\sigma ^2}zw_i-\tau \left( \frac{2}{\sigma }+\frac{1}{\sigma }\right) ^2q_i+\frac{1}{\tau }\left( \frac{\sigma }{\tau }-\tau \left( \frac{2}{\sigma }+\frac{1}{\tau }\right) w_i\right) ^2\right) }{f_{EG}(x_i)}\right. \\&\quad +\left. \frac{\left[ \frac{z_i}{\sigma }\phi (z_i)+\tau f_{EG}\left( \frac{\sigma }{\tau ^2}-\frac{1}{\sigma }\left( z_i+\frac{\sigma }{\tau }\right) w_i\right) \right] ^2}{F_{EG}(x_i)}\right] ,\\ \kappa _{\sigma \alpha }&= \sum _i\frac{\frac{1}{\sigma }z_i\phi (z_i)+\left( \frac{\sigma }{\tau }-\frac{\tau }{\sigma }\left( z_i+\frac{\sigma }{\tau }\right) w_i\right) f_{EG}(x_i)}{F_{EG}(x_i)} \end{aligned}$$

and

$$\begin{aligned} \kappa _{\alpha \alpha }=\frac{n}{\alpha ^2} \end{aligned}$$

where $$w_i(\mu ,\sigma ,\tau )=\frac{\phi \left( \frac{x_i-\mu }{\sigma }-\frac{\sigma }{\tau }\right) }{\Phi \left( \frac{x_i-\mu }{\sigma }-\frac{\sigma }{\tau }\right) }$$ and $$q_i=w_i(\mu ,\sigma ,\tau )\left[ \frac{x_i-\mu }{\sigma }-\frac{\sigma }{\tau }+w_i(\mu ,\sigma ,\tau ) \right]$$.

For large *n*, the observed information matrix $J(\theta )$, converges to the expected information matrix $I(\theta )$. Hence, with the elements of the matrix $J(\theta )$ and the power-normal family being characterised by having a non-singular information matrix [see Pewsey et al. (2012)], it can be concluded that

$$\begin{aligned} \sqrt{n}(\hat{\theta }-\theta )\rightarrow N(\mathbf{0},J^{-1}(\theta )). \end{aligned}$$

That is, the GEG converges to a normal distribution with covariance matrix $J^{-1}(\theta ).$ The standard errors of the estimates of the model parameters can be obtained by calculating the square root of the elements of the diagonal of $\hat{J}^{-1}(\hat{\theta })$. The result of this can be used to find confidence intervals for the model parameters.

## Footnotes

1 This study is perhaps one of the few studies, if not the only one, tackling RTs from a distributional perspective for understanding neuropsychological disorders.

2 Following the work by Lehmann (1953), Durrans (1992) extended the SN distribution for all $\alpha \in {\mathbb{R}}^{+}$ and labelled it the distribution of fractional order statistics. These statistics are also known as AP, Exponentiated, or Generalised Gaussian distributions. These distributions have the PDF $\varphi _F(z;\alpha )=\alpha f(z)\{F(z)\}^{\alpha -1}$, with z $\in {\mathbb{R}}, ~~\alpha \in \mathbb{R}^+$, and where *F* is an absolutely continuous distribution function with probability density function $f(z) =d/dz F(z)$. This new model gives rise to the family of AP (or Exponentiated) distributions, denoted by $Z\sim AP(\alpha )$, which is a new alternative among families of distributions that model high degrees of asymmetry and kurtosis. The AP distribution therefore allows modelling data that do not follow a normal distribution and that rather exhibit high (or low) asymmetry and/or kurtosis.

3 Note that the GEG, ECSN, and SNAP distributions are not yet included in standard GAMLSS R packages such as gamlss.dist and only the specific GAMLSS model required for the analysis of Schledde et al. (2017)’s data set was implemented. While the results reported in this manuscript indicate that it is worthwhile including the GEG distribution in the gamlss.dist R package, the other distributions should be included as well given evidence supporting their value (see references in the main text). Doing so, though, requires more theoretical work on determining several derivatives and other properties of these distributions that are needed for the internal optimisation of parameters. Research on this this matter is under way.

4 Indeed, when both location and scale parameters were modelled, the EG model still provided a better fit (EG model’s AIC = − 78.62 and NO model’s AIC = − 78.59). Having said this, it is important that a GAMLSS model be selected based on its interpretability (Ramires et al. 2021).
